# Neighbor-Neighbor Correlations Explain Measurement Bias in Networks

**DOI:** 10.1038/s41598-017-06042-0

**Published:** 2017-07-17

**Authors:** Xin-Zeng Wu, Allon G. Percus, Kristina Lerman

**Affiliations:** 1 Information Sciences Institute, University of Southern California, Marina del Rey, CA 90292 USA; 2 Department of Physics and Astronomy, University of Southern California, Los Angeles, CA 90089 USA; 3 Institute of Mathematical Sciences, Claremont Graduate University, Claremont, CA 91711 USA

## Abstract

In numerous physical models on networks, dynamics are based on interactions that exclusively involve properties of a node’s nearest neighbors. However, a node’s local view of its neighbors may systematically bias perceptions of network connectivity or the prevalence of certain traits. We investigate the *strong friendship paradox*, which occurs when the majority of a node’s neighbors have more neighbors than does the node itself. We develop a model to predict the magnitude of the paradox, showing that it is enhanced by negative correlations between degrees of neighboring nodes. We then show that by including neighbor-neighbor correlations, which are degree correlations one step *beyond* those of neighboring nodes, we accurately predict the impact of the strong friendship paradox in real-world networks. Understanding how the paradox biases local observations can inform better measurements of network structure and our understanding of collective phenomena.

## Introduction

Local interactions among nodes in a complex network can lead to an astounding array of collective phenomena. Examples include viral outbreaks in social networks, cascading failures in the power grid and financial networks, synchronization of coupled oscillators, opinion dynamics and consensus formation in human groups. Researchers have linked the structure of complex networks to the dynamics of collective phenomena unfolding on them: highly connected nodes amplify viral outbreaks^1–3^, while community structure affects the dynamics of synchronization^4^ and the spread of social contagions^5^.

A node’s own local view of a network, however, may be systematically biased. One source of bias is Feld’s friendship paradox: the number of connections, or degree, of a node is smaller than the average of its neighbor’s degrees^6^. Recently, more subtle forms of the paradox have been proposed. The *strong* friendship paradox^7^ states that the degree of a node tends to be smaller than the *median* of its neighbor’s degrees. Roughly speaking, this is equivalent to the node having fewer neighbors than do a majority of its neighbors. But unlike the original friendship paradox and some recent generalizations^8–11^, the strong friendship paradox does not arise as a straightforward result of sampling from skewed distributions^7^. The strong friendship paradox can dramatically distort local measurements in a network, leading to the “majority illusion”^12^ in which a globally rare attribute may be overrepresented in the local neighborhoods of many nodes. Physical systems whose dynamics are governed by majority rule—from Ising spin interactions^13^ to more complex voting models^14^—may be affected by this paradox.

In this manuscript, we develop a stochastic model to predict the magnitude of the strong friendship paradox. Specifically, we show that (a) increasingly disassortative networks exhibit a larger paradox, and (b) accurately modeling it requires considering degree correlations one step beyond those of neighboring nodes.

Given a network with degree distribution *p*(*k*), we define the global probability of the strong friendship paradox as *P*_paradox_ = ∑_*k*_ *p*(*k*)*f*(*k*), where *f*(*k*) is the probability that a randomly chosen node with degree *k* experiences the paradox. Formally, we define

$$f(k)\equiv P({\rm{Median}}\{{k}_{1}^{^{\prime} },\cdots \,,{k}_{k}^{^{\prime} }\} > k|k),$$

where ${k}_{i}^{^{\prime} }$ is the degree of the node’s *i*th neighbor.

Of course, networks can have structure beyond that given by the degree distribution. The *dK*-series framework^15^ specifies network structure as a series of joint degree distributions of subgraphs of *d* nodes. Thus, a network’s 1*K*-structure is specified by the degree distribution *p*(*k*). The 2*K*-structure captures degree correlations of nodes in connected pairs. This is specified by the joint degree distribution *e*(*k*, *k*′), the probability that an edge links two nodes with degrees *k* and *k*′. It follows that the degree distribution of an edge’s endpoint is $q(k)={\sum }_{k^{\prime} }e(k,k^{\prime} )=kp(k)/\langle k\rangle$. Similarly, a network’s 3*K*-structure is specified by the joint degree distribution of connected triplets, either wedges or triangles. We find that these higher-order degree correlations can be substantial in real-world networks, possibly reflecting their macroscopic organization into a core-periphery structure, and that accounting for them is necessary for a quantitative understanding of the strong friendship paradox.

The strong friendship paradox depends only on the comparison between the degrees of a node and its neighbors. The probability *Q*_>_ that a node sees a neighbor with degree larger than its own can be written as:

1 $${Q}_{ > }=\sum _{k}\sum _{k^{\prime}  > k}P(k^{\prime} |k)p(k)=\langle k\rangle \sum _{k}\sum _{k^{\prime}  > k}\frac{e(k,k^{\prime} )}{k},$$

since the neighbor degree distribution of a degree *k* node is *P*(*k*′|*k*) = *e*(*k*, *k*′)/*q*(*k*). This expression uses information about the network’s 2*K*-structure, which is globally measured by the assortativity coefficient^16^

2 $$r=\frac{1}{{\rm{Var}}(k)}\sum _{k,k^{\prime} }kk^{\prime} [e(k,k^{\prime} )-q(k)q(k^{\prime} )],$$

where the variance of *k* is taken with respect to the distribution *q*(*k*):

$${\rm{Var}}(k)=\sum _{k}{k}^{2}q(k)-{[\sum _{k}kq(k)]}^{2}$$

In assortative networks (*r* > 0), nodes preferentially link to other nodes with similar degree, while in disassortative networks (*r* < 0), they prefer to link to others with dissimilar degree, e.g., high to low degree nodes. Since *k* is in the numerator of the sum for *r* but in the denominator of Eq. (1), given the normalization ${\sum }_{k,k^{\prime} }e(k,k^{\prime} )=1$, we may expect disassorativity to magnify the paradox in networks, and assortativity to suppress it. Previous numerical results for the conventional friendship paradox^10^ support this prediction.

## Results

### The 2*K* model

Given a randomly chosen node with degree *k*, define an indicator function *x*_*i*_, *i* = 1…*k*, to track the degree of the node’s *i*th neighbor:

3 $${x}_{i}={{\bf{1}}}_{{k}_{i}^{^{\prime} } > k}=\{\begin{array}{cc}1 & {\rm{if}}\,{k}_{i}^{^{\prime} } > k\\ 0 & {\rm{if}}\,{k}_{i}^{^{\prime} }\le k\end{array}$$

To a close approximation (and exactly, for odd *k*), the node is in the paradox regime if $\bar{x}\equiv \frac{1}{k}{\sum }_{i=1}^{k}{x}_{i} > \frac{1}{2}$.

To understand how network structure affects the strong friendship paradox, we now examine *μ*_*x*_(*k*), the probability that a neighbor (say the *i*th one) of a randomly chosen degree-*k* node has degree greater than *k*:

4 $${\mu }_{x}(k)=P({x}_{i}=\mathrm{1|}k)=P({k}_{i}^{^{\prime} } > k|k)=\sum _{k^{\prime}  > k}\frac{e(k,k^{\prime} )}{q(k)}$$

If we assume that degrees of neighbors are independent and identically distributed random variables, the probability for a degree-*k* node to observe the strong friendship paradox is then given by the binomial distribution:

5 $$\begin{array}{rcl}f(k) & = & P(\bar{x} > \frac{1}{2})=P(\sum _{i\mathrm{=1}}^{k}{x}_{i} > \frac{k}{2})\\  & = & \sum _{i=\lceil \frac{k+1}{2}\rceil }^{k}(\begin{array}{c}k\\ i\end{array}){\mu }_{x}{(k)}^{i}{\mathrm{[1}-{\mu }_{x}(k)]}^{k-i}\mathrm{.}\end{array}$$

For large *k*, *f*(*k*) is close to Gaussian. In terms of the normal distribution’s cumulative distribution function Φ,

6 $$f(k)\approx 1-{\rm{\Phi }}\{\frac{1-2{\mu }_{x}(k)}{2\sqrt{{\mu }_{x}(k\mathrm{)[1}-{\mu }_{x}(k)]}}\sqrt{k}\}\mathrm{.}$$

To demonstrate how assortativity modifies the strong friendship paradox, we consider a network with *e*(*k*, *k*′) that has a bivariate log-normal distribution, a long-tailed distribution defined on positive domain of *k*, with equal means *m*, equal variances *s* ^2^, and correlation coefficient *c*. This form of the distribution allows for analytical treatment of the problem. Thus, the assortativity can be written as

7 $$r=\frac{{\rm{Cov}}(k,k^{\prime} )}{{\rm{Var}}(k)}=\frac{{e}^{c{s}^{2}}-1}{{e}^{{s}^{2}}-1}\mathrm{.}$$

Note that the assortativity is bounded by $-{e}^{-{s}^{2}}\le r\le 1$, and increases with *c*. We can then express *μ*_*x*_(*k*) analytically as

8 $$\begin{array}{rcl}{\mu }_{x}(k) & = & P(k^{\prime}  > k|k)=P(\mathrm{log}k^{\prime}  > \,\mathrm{log}\,k|k)\\  & = & 1-{\rm{\Phi }}\{\frac{\mathrm{log}\,k-E(\mathrm{log}\,k^{\prime} |\,\mathrm{log}\,k)}{\sqrt{{\rm{Var}}(\mathrm{log}\,k^{\prime} |\,\mathrm{log}\,k)}}\}\\  & = & 1-{\rm{\Phi }}\{\frac{\mathrm{(1}-c)(\mathrm{log}\,k-m)}{\sqrt{\mathrm{(1}-{c}^{2}){s}^{2}}}\}\\  & = & 1-{\rm{\Phi }}\{\frac{\mathrm{log}\,k-m}{s}\sqrt{\frac{1-c}{1+c}}\}\mathrm{.}\end{array}$$

**Figure 1:**
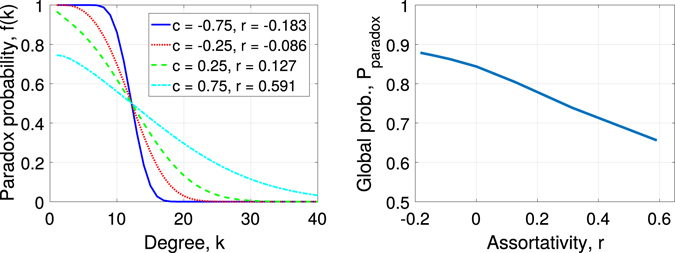
Impact of assortativity on strong friendship paradox. The matrix *e*(*k*, *k*′) has bivariate log-normal distribution with parameters *m* = 2.5, *s* = 1.25, *c* ranges from −0.75 to 0.75, which corresponds to assortativity *r* in the range −0.18 to 0.6.

It follows that *f*(*k*) decreases with *k*. As the network becomes more disassortative (*c* < 0), *f*(*k*) undergoes an increasingly sharp transition from 1 to 0 around *k* = *e* ^*m*^ (Fig. 1(a)). Given that most nodes have low degree, this leads to a globally stronger paradox in more disassortative networks (Fig. 1(b)), consistent with our prediction.

The structure of real-world networks creates conditions for the paradox. Table 1 reports the observed fraction of nodes in these networks who see a majority of their neighbors with a larger degree. This fraction is very large in all networks, ranging from 75% to 90%.

**Table 1:** Observed fraction of nodes in real-world networks that experience the strong friendship paradox, compared to predictions of the two proposed models.

Network	Type	Observed	3K model	2K model
LiveJournal	Social	83.71%	84.43%	86.95%
Youtube	Social	89.94%	88.51%	90.34%
Skitter	Internet	88.62%	90.35%	95.79%
Google	Web	77.31%	78.25%	84.36%
ArXiv HEP	Citation	78.71%	79.67%	83.83%
English words	Semantic	75.23%	71.00%	71.05%

**Figure 2:**
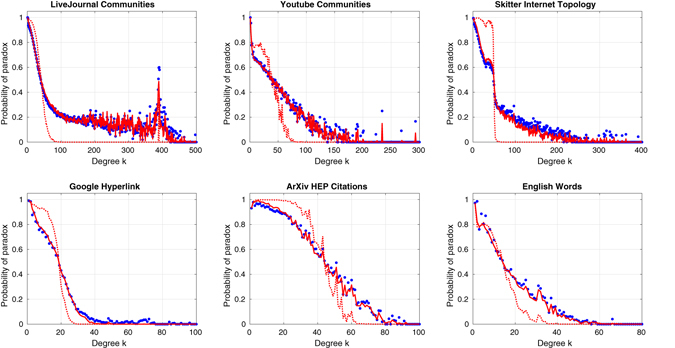
Probability of the strong friendship paradox in six real-world networks, comparing observed fraction of degree-*k* nodes that are in the paradox regime (blue dots) to predictions of the 2*K* model (dotted red line) and the 3*K* model (solid red line).

Table 1 shows that the observed fractions of nodes experiencing the paradox are close to the global probabilities predicted by the 2*K* model, when *μ*_*x*_(*k*) is set to the actual frequency with which a neighbor of a degree-*k* node has larger degree. However, a breakdown by degree class reveals significant deviations. Figure 2 plots the paradox probability *f*(*k*) for a degree-*k* node (blue dots). We define the degree at which the 2*K* estimate (Eq. (6)) of paradox probability is 0.5 as the critical degree *k*_*c*_ of the network. By construction, *k*_*c*_ = Median(*q*(*k*)). Nodes with degree *k* < *k*_*c*_ are likely to experience the paradox, while those with *k* > *k*_*c*_ are unlikely to do so. The 2*K* model (dotted line) overestimates the paradox for low-degree nodes and underestimates it for high-degree nodes. This suggests that the 2*K* model is insufficient, and we need to take into account structure beyond degree correlations of connected pairs of nodes.

### The 3*K* model

If neighbor degrees are identically distributed but *correlated* random variables, Eq. (6) must be modified to represent a multivariate rather than a single binomial distribution. To deal with the correlation, we now consider a pair of neighbors, with degree *k*_*i*_ and *k*_*j*_, of a single degree-*k* node, and their indicator functions *x*_*i*_ and *x*_*j*_ as defined in Eq. (3). The corresponding multivariate normal approximation then gives

9 $$f(k)=1-{\rm{\Phi }}\{\frac{\frac{1}{2}-{\mu }_{x}(k)}{{\sigma }_{x}(k)}\},$$

where the variance ${\sigma }_{x}^{2}(k)$ is now

10 $$\begin{array}{rcl}{\sigma }_{x}^{2}(k) & = & {\rm{Var}}(\bar{x})={\rm{Var}}(\frac{1}{k}\sum _{i=1}^{k}{x}_{i})\\  & = & \frac{1}{{k}^{2}}[\sum _{i=1}^{k}{\rm{Var}}({x}_{i})+2\sum _{i=1}^{k-1}\sum _{j=i+1}^{k}{\rm{Cov}}({x}_{i},{x}_{j})]\\  & = & \frac{1}{k}{\mu }_{x}(k\mathrm{)[1}-{\mu }_{x}(k)]+\frac{k-1}{k}\,{\rm{Cov}}({x}_{i},{x}_{j}\mathrm{).}\end{array}$$

Unlike in Eq. (6), where *f*(*k*) is completely determined by *μ*_*x*_(*k*), the 3*K* model requires the covariance term to be specified. Using values determined empirically from real-world networks as in the 2K model, we obtain very accurate paradox probability estimates (solid line in Fig. 2). These estimates also improve on the global 2K results shown in Table 1 for all cases except Youtube and English words, where the two estimates are nearly identical due to their close agreement for low degree values that represent a large fraction of nodes in the network.

To understand the effect of the covariance term, consider the 3*K*-distribution $t({k}_{i}^{^{\prime} },k,{k}_{j}^{^{\prime} })$, the joint degree distribution of a connected ordered triplet of nodes with degrees $({k}_{i}^{^{\prime} },k,{k}_{j}^{^{\prime} })$. Conditioning on the degree *k* of the focal node gives the joint degree distribution of its two neighbors:

$$P({k}_{i}^{^{\prime} },{k}_{j}^{^{\prime} }|k)=P({k}_{i}^{^{\prime} },k,{k}_{j}^{^{\prime} }|k)=\frac{t({k}_{i}^{^{\prime} },k,{k}_{j}^{^{\prime} })}{g(k)}$$

11 $$g(k)=\sum _{{k}_{i}^{^{\prime} },{k}_{j}^{^{\prime} }}t({k}_{i}^{^{\prime} },k,{k}_{j}^{^{\prime} }\mathrm{).}$$

The indicator function covariance term in Eq. (10) is

12 $$\begin{array}{rcl}{\rm{Cov}}({x}_{i},{x}_{j}) & = & P({x}_{i}=1,{x}_{j}=\mathrm{1|}k)-P{({x}_{i}=1|k)}^{2}\\  & = & P({k}_{i}^{^{\prime} } > k,{k}_{j}^{^{\prime} } > k|k)-P{({k}_{i}^{^{\prime} } > k|k)}^{2},\end{array}$$

where

13 $$P({k}_{i}^{^{\prime} } > k,{k}_{j}^{^{\prime} } > k|k)=\frac{1}{g(k)}\sum _{{k}_{i}^{^{\prime} } > k,{k}_{j}^{^{\prime} } > k}t({k}_{i}^{^{\prime} },k,{k}_{j}^{^{\prime} }\mathrm{).}$$

and $P({k}_{i}^{^{\prime} } > k|k)$ is given by Eq. (4). Thus, the covariance takes into account correlations only up to the level of chains $({k}_{i}^{^{\prime} },k,{k}_{j}^{^{\prime} })$. Any higher-order correlations beyond 3*K*, such as those involving connected subgraphs of four nodes, would no longer be consistent with a normal approximation for *f*(*k*), since they would involve information beyond the second moment of the indicator function. The remarkable success of the 3*K* model in Fig. 2 suggests that such higher-order correlations are not needed to explain the paradox, or that they are negligible in real-world networks.

Define the neighbor-neighbor correlation as

14 $${\rho }_{x}(k)=\frac{{\rm{Cov}}({x}_{i},{x}_{j})}{\sqrt{{\rm{Var}}({x}_{i}){\rm{Var}}({x}_{j})}}=\frac{{\rm{Cov}}({x}_{i},{x}_{j})}{{\mu }_{x}(k\mathrm{)[1}-{\mu }_{x}(k)]}\mathrm{.}$$

**Figure 3:**
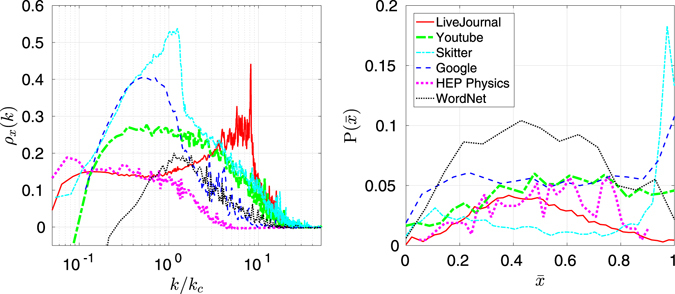
(Left) Neighbor-neighbor correlation coefficient by degree class for each network discussed in this paper. (Data have been smoothed). (Right) Distribution of $\bar{x}$ at the critical degree *k*_*c*_.

Note that this correlation, like *σ*_*x*_(*k*), is based not on the neighbors’ degrees but on the indicator function comparing them to the node’s degree. Figure 3 shows empirically determined values of *ρ*_*x*_(*k*) for the real-world networks we studied. Recall that in the 2*K* model, the probability that a degree-*k* node has a neighbor with degree greater than *k* is determined completely by *e*(*k*, *k*′) and is unrelated to the degrees of the other neighbors. One might reasonably expect low-degree nodes to have mostly neighbors of higher degree, high-degree nodes to have mostly neighbors of lower degree, and medium-degree nodes to have a mix of both. Figure 3, however, depicts a different scenario: medium-degree nodes prefer to have neighbors with similar degree *to one another*—whether those neighbors have higher or lower degree. To see how these correlations may be indicative of the macroscopic organization of a network, we plot the distribution of $\bar{x}$, the fraction of higher-degree neighbors, for nodes with *k* = *k*_*c*_. In the technological networks of Skitter and Google, such medium-degree nodes link more often to high-degree nodes, possibly reflecting a hierarchical network structure with medium-degree at the top level and high-degree nodes at the next level. The remaining networks show a broad distribution of $\bar{x}$, consistent with a core-periphery network structure where medium-degree nodes link to higher-degree nodes in the core and to lower-degree nodes in the periphery^17, 18^.

## Discussion

The connection between local measurement bias and network structure revealed by the strong friendship paradox is crucial for several reasons. It is often impractical to observe large networks in their entirety: instead, researchers estimate network properties by exploring local neighborhoods of select nodes. The paradox, however, may systematically bias local views of networks structure, including sampled degree distribution^19^. The strong friendship paradox also affects measurements of information in networks. Consider a network where nodes have attributes and estimate their prevalence from local observations. When attribute and degree are correlated, the paradox can create an illusion that the attribute is common even when it is globally rare^12^. Finally, quantifying measurement bias may be necessary for predicting the evolution of dynamic processes such as domain formation by majority rule in interacting spin systems^13^, or synchronization of frequencies in complex networks such as electrical power grids^20^. Accounting for neighbor-neighbor correlations could be instrumental to the success of network models for such systems.

In this paper, we have studied strong friendship paradox in networks, a phenomenon that distorts nodes’ observations of local network structure. The paradox leads most nodes to observe that a majority of their neighbors have a larger degree than their own. We have developed an analytical model of the strong friendship paradox, enabling highly accurate predictions of its strength in networks. In contrast to Feld’s friendship paradox^6^, which exists in any network with variance in the degree distribution, the strong friendship paradox requires information about higher-order network structure. Specifically, negative correlations between degrees of connected nodes—given by network’s 2*K* structure—will magnify the paradox, especially in networks with a skewed degree distribution. The impact of disassortativity, however, is modulated by degree correlations between nodes’ neighbors. These correlations—given by network’s 3*K* structure—are necessary to accurately quantify the paradox. The success of the 3*K* model in explaining the paradox is consistent with the observation^15^ that it is sufficient to capture known network properties. In order to mitigate the effects of local measurement bias in networks, it is important to account for the strong friendship paradox and how it is impacted by higher-order network structure.

## Methods

### Data description

We study six networks from a variety of domains, including social networks (friendship links on LiveJournal blogging site soc-LiveJournal1^21^, community structure on Youtube com-Youtube^21^) technological networks (Skitter internet graph as-skitter^21^ and Google web hyperlink graph web-Google^21^), scientific citations graph (Arxiv cit-HepPh^21^), and relationships between English words^22^. Table 2 shows some basic properties of the networks. These networks vary in size from 34.5 K nodes (Arxiv) to almost 4 M nodes (LiveJournal), and assortativity from 0.045 (LiveJournal) to −0.08 (Skitter).

**Table 2:** List of real world networks and their basic profiles.

	Name	Type	Nodes	Edges	Assortativity
1	LiveJournal	Social	3,997,962	34,681,189	0.045145
2	Youtube	Social	1,134,890	2,987,624	−0.036910
3	Skitter	Internet	1,696,415	11,095,298	−0.081422
4	Google	Hyperlink	875,713	4,322,051	−0.055089
5	ArXiv HEP	Citation	34,546	420,877	−0.005943
6	WordNet	Semantic	146,005	656,999	−0.006286

Note that directed edges, if they exist, are treated as undirected edges.
