# Author Correction: Neighbor-Neighbor Correlations Explain Measurement Bias in Networks

**DOI:** 10.1038/s41598-018-36018-7

**Published:** 2018-12-04

**Authors:** Xin-Zeng Wu, Allon G. Percus, Kristina Lerman

**Affiliations:** 1 Information Sciences Institute, University of Southern California, Marina del Rey, CA 90292 USA; 2 Department of Physics and Astronomy, University of Southern California, Los Angeles, CA 90089 USA; 3 Institute of Mathematical Sciences, Claremont Graduate University, Claremont, CA 91711 USA

Correction to: *Scientific Reports* 10.1038/s41598-017-06042-0, published online 17 July 2017

The Acknowledgements section in this Article was omitted and should read:

“This work was funded in part by Army Research Office under contract W911NF-16-1-0306 and by the National Science Foundation under grant SMA-1360058.”

